# Hyaluronic acid enhances the effect of the PAMPS/PDMAAm double-network hydrogel on chondrogenic differentiation of ATDC5 cells

**DOI:** 10.1186/1471-2474-15-222

**Published:** 2014-07-06

**Authors:** Nobuto Kitamura, Takayuki Kurokawa, Takaaki Fukui, Jian P Gong, Kazunori Yasuda

**Affiliations:** 1 Department of Sports Medicine and Joint Surgery, Graduate School of Medicine, Hokkaido University, Kita-15, Nishi-7, Kita-ku, Sapporo 060-8638, Japan; 2 Laboratory of Soft and Wet Matter, Department of Advanced Transdisciplinary Sciences, Faculty of Advanced Life Science, Hokkaido University, Sapporo, Japan

**Keywords:** Hyaluronic acid, Chondrogenic differentiation, ATDC5, Double-network hydrogel, Polymer

## Abstract

**Background:**

A double-network (DN) gel, which was composed of poly-(2-Acrylamido-2-methylpropanesulfonic acid) and poly-(N,N’-dimethyl acrylamide) (PAMPS/PDMAAm), has the potential to induce chondrogenesis both in vitro and in vivo. The present study investigated whether DN gel induced chondrogenic differentiation of ATDC5 cells in a maintenance medium without insulin, and whether supplementation of hyaluronic acid enhanced the chondrogenic differentiation effect of DN gel.

**Methods:**

ATDC5 cells were cultured on the DN gel and the polystyrene (PS) dish in maintenance media without insulin for 21 days. Hyaluronic acid having a molecular weight of approximately 800 kDa was supplemented into the medium so that the concentration became 0.01, 0.1, or 1.0 mg/mL. The cultured cells were evaluated using immunocytochemistry for type-2 collagen and real time PCR for gene expression of type-2 collagen, aggrecan, and Sox9 at 7 and 21 days of culture.

**Results:**

The cells cultured on the DN gel formed nodules and were stained with an anti-type-2 collagen antibody, and expression of type-2 collagen and aggrecan mRNA was significantly greater on the DN gel than on the PS dish surface (p < 0.05) in the hyaluronic acid-free maintenance medium. Hyaluronic acid supplementation of a high concentration (1.0 mg/mL) significantly enhanced expression of type-2 collagen and aggrecan mRNA in comparison with culture without hyaluronic acid at 21 days (p < 0.05).

**Conclusions:**

The DN gel induced chondrogenic differentiation of ATDC5 cells without insulin. This effect was significantly affected by hyaluronic acid, depending on the level of concentration. There is a high possibility that hyaluronic acid plays an important role in the in vivo hyaline cartilage regeneration phenomenon induced by the DN gel.

## Background

Great attention to treating damaged joint surfaces has arisen from a growing need to repair damaged articular cartilage. Therefore, much effort has been made to develop tissue-engineered constructs for articular cartilage repair. We have recently demonstrated that articular cartilage repair is feasible using a synthetic PAMPS/PDMAAm double-network (DN) hydrogel, which consists of poly-(2-acrylamido-2-methylpropanesulfonic acid) (PAMPS) and poly-(N,N’-dimethyl acrylamide) (PDMAAm), without using tissue-engineered cartilage-like tissue or a cell-seeded material in an animal cartilage defect model [1-3]. To clarify a part of the mechanism of this effect induced by the PAMPS/PDMAAm DN gel, we have studied the in vitro behavior of the ATDC5 cells on synthetic hydrogels [1,4,5]. Previous study showed that the PAMPS/PDMAAm DN gel significantly enhances chondrogenic differentiation of ATDC5 cells in a differentiation medium with insulin [1]. Further, the single-network PAMPS gel, which is a component gel of the DN gel, differentiates chondrogenic ATDC5 cells into chondrocytes even in an insulin-free medium [4]. These results imply that chondrogenic differentiation of ATDC5 cells is induced by the culture microenvironment modified by gel components as well as insulin supplementation. For example, Engler et al. [6] reported that the elasticity of the substrate on which the cultured cells are attached directs stem cell differentiation. However, it is not known whether the PAMPS/PDMAAm DN gel induces chondrogenic differentiation of ATDC5 cells in a maintenance medium without insulin.

In addition, we have reported that supplementation of hyaluronic acid significantly enhanced the chondrogenic differentiation of ATDC 5 cells cultured on PAMPS gel [5]. This finding is important as are previous reports demonstrating that hyaluronic acid enhances chondrogenesis [7-9]. However, PAMPS gel is not suitable for clinical applications in its present form from biomechanical and biological considerations. First, the PAMPS gel is biomechanically much weaker than the PAMPS/PDMAAm DN gel. The PAMPS/PDMAAm DN gel shows a fracture strength as high as 3.1 MPa, whereas the fracture strength of the single-network gels ranged a few to several hundreds of kilopascals [10,11]. Secondly, previous in vivo study has demonstrated that although the PAMS gel induces chondrogenesis in vivo, the degree of cartilage repair is significantly better with PAMPS/PDMAAm DN gel than with PAMPS gel [12]. Therefore, it is of importance to clarify the in vitro influence of hyaluronic acid on the chondrogenic differentiation induced by the PAMPS/PDMAAm DN gel as it is possible that hyaluronic acid may enhance cartilage regeneration.

The purposes of this study were to evaluate whether PAMPS/PDMAAm DN gel induces chondrogenic differentiation of ATDC5 cells in a maintenance medium without insulin, and whether supplementation of hyaluronic acid into the maintenance medium enhances the chondrogenic differentiation effect of the PAMPS/PDMAAm DN gel on the ATDC5 cells.

## Methods

### Preparation of the DN gel

The PAMPS/PDMAAm DN gel was synthesized using the previously reported two-step sequential polymerization method [11]. 2-acrylamido-2-methyl-1-propanesulfonic acid (AMPS) (Toagosei Co. Ltd., Japan) and N,N’-dimethyl acrylamide (DMAAm) (KOHJIN Co., Ltd., Tokyo, Japan) were used as purchased. Briefly, PAMPS hydrogel was obtained by radical polymerization using N,N^’^-methylenebisacrylamide (MBAA) (Tokyo Chemical Industry Co., Ltd., Tokyo, Japan) as a cross-linker and 2-oxoglutaric acid (Wako Pure Chemical Industries, Ltd, Osaka, Japan) as an initiator. The monomer concentration was 1 mol/l for PAMPS, 4 mol% for the cross-linker, and 0.1 mol% for the initiator. Aqueous solution containing a monomer, cross-linker, and the initiator was injected into a cell consisting of a pair of glass plates separated by a silicone rubber. The cell was irradiated with ultraviolet (UV) light (wave length 365 nm) for about 8 hours under argon gas atmosphere. The DN gel was synthesized by the sequential network formation technique (two-step method). The PAMPS hydrogel (1^st^ network) was immersed in an aqueous solution of 2 mol/L DMAAm, containing 0.1 mol% MBAA, and 0.1 mol% 2-oxoglutaric acid for one day until reaching the equilibrium. The 2^nd^ network (PDMAAm) was subsequently polymerized in the presence of the PAMPS hydrogel by irradiating UV for 8 hours between two plates of glasses under argon gas atmosphere. After polymerization, the PAMPS/PDMAAm DN gel was immersed in 0.9 % NaCl solution for 1 week and the water was changed twice daily to remove any un-reacted materials. After sterilizing by autoclaving (120 degrees Celsius, 20 min), gel disks were punched out of a gel plate with a hole puncher having a diameter of 1.5 cm. The gel disks were then placed in a 24-well polystyrene (PS) tissue culture dish for cell culture.

### Cell culture

The ATDC5 cell line was obtained from the RIKEN cell bank (Tsukuba, Japan).The cells were cultured in the maintenance medium consisting of a 1:1 mixture of Dulbecco’s modified Eagle’s medium and Ham’s F-12 medium (Life Technologies, GIBCO, Carlsbad, CA, USA) supplemented with 5% fetal bovine serum, 10 mg/ml human transferrin (Roche Applied Science, Indianapolis, IN, USA) and 3 × 10^−8^ M sodium selenite (Sigma–Aldrich, St. Louis, MO, USA). The ATDC5 cells were seeded at a cell density of 5 × 10^4^ cells/cm^2^, and cultured on the PAMPS/PDMAAm DN gel and the PS dish surface at 37°C under 5% CO2 for 21 days. The PS dish was used as the control. The maintenance medium was changed twice each week without damaging the gels.

### Study design

This study was composed of 2 sub-studies. The first sub-study was conducted to test the hypothesis that the PAMPS/PDMAAm DN gel may induce chondrogenic differentiation of ATDC5 cells even in a maintenance medium without insulin. We compared the ATDC5 cells cultured on the DN gel with the cells cultured on the PS dish (n = 6, each), using the maintenance medium. In the second sub-study, we tested the hypothesis that supplementation of hyaluronic acid into the maintenance medium may enhance the chondrogenic differentiation effect of the PAMPS/PDMAAm DN gel on the ATDC5 cells. We prepared 3 types of the maintenance medium containing hyaluronic acid (ARTZ, Seikagaku Co., Tokyo, Japan) at the concentration of 0.01, 0.1, or 1 mg/mL, respectively. The hyaluronic acid had a molecular weight of approximately 800 kDa. The ATDC5 cells cultured in the 3 hyaluronic acid-supplemented media were compared with the cells cultured in the maintenance medium without hyaluronic acid (n = 6, each). In each sub-study, we performed real-time polymerase chain reaction (PCR) analyses to evaluate expression of type-2 collagen, aggrecan, and Sox9 mRNA in the cultured ATDC5 cells at 7 and 21 days of culture. We also carried out immunocytochemical examination to assess expression of type-2 collagen at the same periods (n = 2, each).

### Evaluation methods

#### Real time PCR

Total RNA was isolated from the ATDC5 cells using the RNeasy mini kit (Qiagen Inc., Valencia, CA). RNA quality from each sample was assured by the A260/280 absorbance ratio. The RNA (100 ng) was reverse-transcribed into single strand cDNA using PrimeScript® RT reagent kit (TakaraBio, Ohtsu, Japan). Real time PCR reactions for type-2 collagen, aggrecan, Sox9, and glyceraldehyde-3-phosphate dehydrogenase (GAPDH), were conducted using the SYBR green system (TakaraBio, Ohtsu, Japan). The sequences of primers used in real time PCR analyses were as follows: type-2 collagen forward AGGGCAACAGCAGGTTCACATAC; reverse TGTCCACACCAAATTCCTGTTCA. Aggrecan forward AGTGGATCGGTCTGAATGACAGG; reverse AGAAGTTGTCAGGCTGGTTTGGA. Sox9 forward CAGTACCCGCATCTGCAC; reverse TCTCTTCTCGCTCTCGTT. GAPDH forward TGTGTCCGTCGTGGATCTGA; reverse TTGCTGTTGAAGTCGCAGGAG. The expression level of the gene was normalized to GAPDH.

### Immunocytochemical examination

Cells were fixed with 4% paraformaldehyde in phosphate-buffered saline without calcium (PBS(−)), and permeabilized with 0.1% Triton X-100 in PBS, followed by pretreatment to block nonspecific reactions with 5% nonimmune goat serum in PBS(−). For collagen staining, the primary immunoreaction was carried out with a mouse monoclonal antibody against type-2 collagen (Abcam Inc., Cambridge, MA, USA). The secondary immunoreaction was carried out with Alexa 488-conjugated goat anti-mouse IgG (Invitrogen, Carlsbad, CA, USA) in 1% nonimmune goat serum in PBS, followed by rinsing with PBS(−). For cell nucleus staining, cells were incubated on 1 μg/ml Hoechst 33258 (Dojindo, Kumamoto, Japan) for 1 min, followed by rinsing with PBS(−). Fluorescent images were recorded with a fluorescence microscope.

### Statistical analysis

All data were described as the mean and standard deviation values. A commercially available software program (StatView 5.0, SAS Institute Inc., Cary, NC, USA) was used for statistical calculation. To test the first hypothesis, we compared each gene expression in the maintenance medium without hyaluronic acid between cultures on the PS dish and the DN gel at each period, using unpaired t-test. To test the second hypothesis, we compared each gene expression among the cells cultured on the DN gel in the maintenance medium with or without hyaluronic acid, using two-way analysis of variance (ANOVA). This was followed by Dunnett’s test for post-hoc multiple comparisons at each period if ANOVA detected statistical significance. The significance level was set at p = 0.05 in each comparison.

## Results

### The effect of the PAMPS/PDMAAm DN gel on chondrogenic differentiation of ATDC5 cells

**Figure 1:**
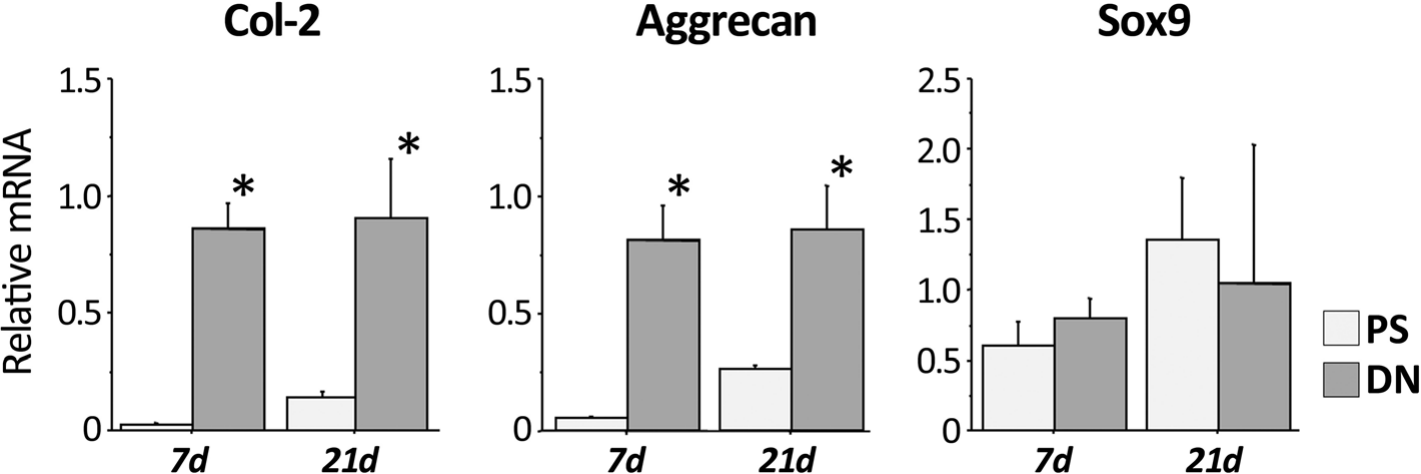
**Gene expression of type-2 collagen (Col-2), aggrecan, and Sox9 in ATDC5 cells cultured on the PAMPS/PDMAAm double-network (DN) gel and the polystyrene (PS) dish at 7 and 21 days of culture.** Expression of type-2 collagen and aggrecan genes was significantly greater on the DN gel than on the PS dish surface. *p < 0.05 vs. PS dish at each period.

**Figure 2:**
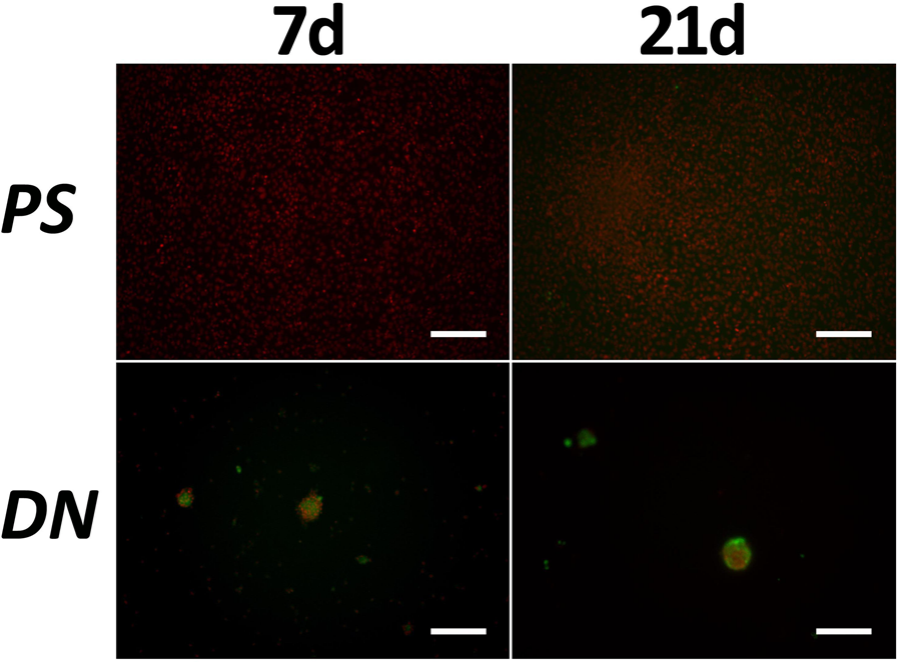
**Expression of type-2 collagen in ATDC5 cells cultured on the PAMPS/PDMAAm double-network (DN) gel and the polystyrene (PS) dish at 7 and 21 days of culture.** The cells cultured on the DN gel formed nodules and were stained with an anti-type-2 collagen antibody (green). White scale bars show a length of 200 micrometers.

At 7 days, mRNA expression of type-2 collagen and aggrecan was significantly greater in culture on the DN gel than in culture on the PS dish (p < 0.0001 and p < 0.0001, respectively) (Figure 1). At 21 days, mRNA expression of type-2 collagen and aggrecan was significantly greater in culture on the DN gel than in culture on the PS dish (p = 0.0008 and p = 0.0008, respectively) (Figure 1). There were no significant differences in mRNA expression of Sox9 between the 2 culture conditions at each period. In immunocytochemical examination performed at 21 days, the cells cultured on the DN gel surface formed nodules and type-2 collagen was expressed in the nodules (Figure 2), while we did not find nodule formation or type-2 collagen expression on the PS dish.

### The influence of hyaluronic acid supplementation on the chondrogenic differentiation effect of the PAMPS/PDMAAm DN gel on the ATDC5 cells

**Figure 3:**
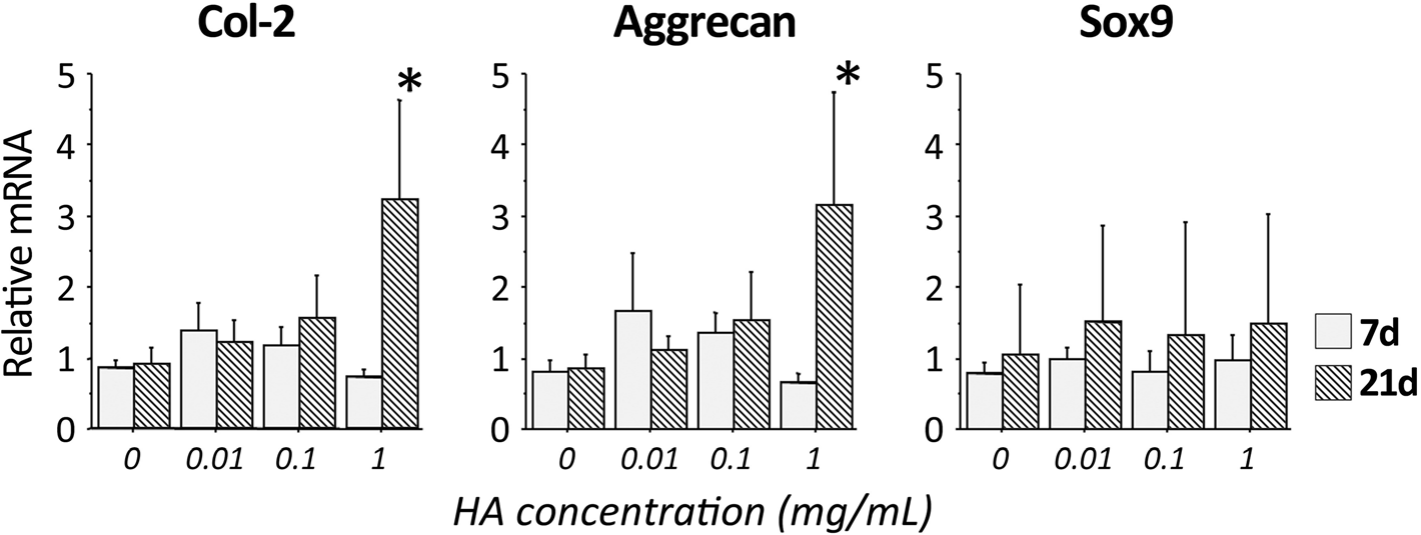
**Gene expression of type-2 collagen (Col-2), aggrecan, and Sox9 in ATDC5 cells cultured on the PAMPS/PDMAAm double-network (DN) gel treated with different concentration of hyaluronic acid (HA) at 7 and 21 days of culture.** *p < 0.05 vs. 0 mg/mL HA at each period (Dunnett’s test).

**Figure 4:**
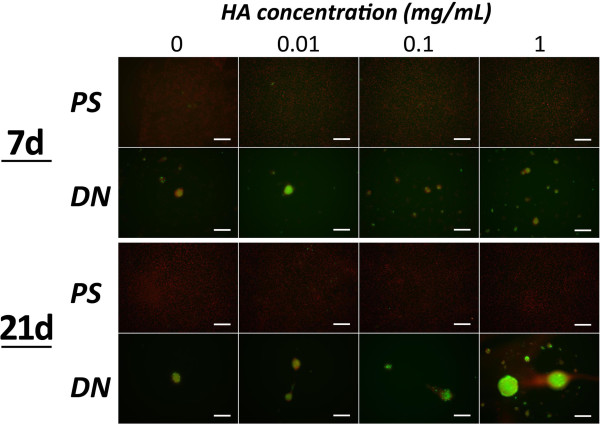
**Expression of type-2 collagen in ATDC5 cells cultured with different concentration of hyaluronic acid (HA) at 7 and 21 days of culture.** At 21 days, type-2 collagen was markedly expressed in nodules cultured on the DN gel surface in the medium supplemented by the high concentration (1.0 mg/mL) of HA. PS: polystyrene dish, DN: PAMPS/PDMAAm double-network gel. White scale bars show a length of 200 micrometers.

Concerning expression of type-2 collagen mRNA, the ANOVA demonstrated significant effects of the degrees of hyaluronic acid concentration (p = 0.0088) and the time (p = 0.0027) (Figure 3). Expression of type-2 collagen mRNA increased, depending on the concentration of hyaluronic acid. Hyaluronic acid supplementation of a high concentration (1.0 mg/mL) significantly enhanced expression of type-2 collagen mRNA in comparison with culture without hyaluronic acid supplementation at 21 days, while we did not find any effect of hyaluronic acid supplementation at 7 days. In immunocytochemical examination at 21 days, type-2 collagen was markedly expressed in nodules cultured on the DN gel surface in the medium supplemented by the high concentration (1.0 mg/mL) of hyaluronic acid (Figure 4).Regarding mRNA expression of aggrecan, the ANOVA also showed a significant effects of the degrees of hyaluronic acid concentration (p = 0.0411) and the time (p = 0.0380) (Figure 3). Expression of aggrecan mRNA increased, depending on the concentration of hyaluronic acid. Hyaluronic acid supplementation of a high concentration (1.0 mg/mL) significantly enhanced expression of aggrecan mRNA in comparison with culture without hyaluronic acid supplementation at 21 days, while we did not find any effect of hyaluronic acid supplementation at 7 days.Concerning mRNA expression of Sox9, the ANOVA did not indicate any significance (Figure 3). In addition, supplementation of hyaluronic acid into the medium did not provide any significant effects on the ATDC5 cells cultured on the PS dish.

## Discussion

The first sub-study demonstrated that mRNA expression of type-2 collagen and aggrecan was significantly greater in culture on the DN gel than in culture on the PS dish at 7 and 21 days. In addition, the immunocytochemical examination performed at 21 days showed that cells cultured on the DN gel surface formed nodules and that type-2 collagen was expressed in the nodules, while we did not find nodule formation or type-2 collagen expression on the PS dish. These results showed that the PAMPS/PDMAAm DN gel induced chondrogenic differentiation of ATDC5 cells even in a maintenance medium without insulin. The second sub-study showed that expression of type-2 collagen and aggrecan mRNAs increased with time, depending on the concentration of hyaluronic acid. The expression was remarkable in the medium containing hyaluronic acid of a high concentration (1.0 mg/mL) at 21 days. In addition, immunocytochemical examination showed that the remarkable expression of type-2 collagen was confirmed in a protein level at 21 days in the cells cultured on the DN gel surface in the medium supplemented with the high concentration (1.0 mg/mL) of hyaluronic acid. These results demonstrated that supplementation of hyaluronic acid into the maintenance medium enhanced the chondrogenic differentiation effect of the PAMPS/PDMAAm DN gel on the ATDC5 cells. We did not find any effects of hyaluronic acid supplementation of the cells cultured on the PS dishes. Therefore, we should note that hyaluronic acid indirectly influences the chondrogenic differentiation of the ATDC5 cells, and that hyaluronic acid enhances the chondrogenic differentiation effect of the PAMPS/PDMAAm DN gel on the ATDC5 cells.

Previous studies reported that the PAMPS/PDMAAm DN gel has an ability to induce chondrogenesis in vivo [1-3,12]. However, this is the first study that the synthetic PAMPS/PDMAAm DN gel has an ability to induce chondrogenesis in vitro without existence of insulin. As for the mechanism of this amazing ability of the PAMPS/PDMAAm DN gel, Kwon et al. [4] reported that the single-network PAMPS gel can induce chondrogenic differentiation of the ATDC5 cells in vitro in the maintenance medium without insulin and the single-network PDMAAm gel can induce chondrogenic differentiation of the ATDC5 cells in the presence of insulin. This is an important observation because these single-network gels are components of the PAMPS/PDMAAm DN gel. They speculated that the surface electrostatic property of the gel affects chondrogenic differentiation of ATDC5 cells as well as physical conditions created by the mechanical properties of the gel surface. Therefore, it is possible that the PAMPS/PDMAAm DN gel added the chondrogenic ability of these gels in itself.

Concerning the effect of hyaluronic acid, the present study demonstrated that the in vitro induction effect of the PAMPS/PDMAAm DN gel on chondrogenic differentiation of ATDC5 cells was affected by hyaluronic acid, depending on the level of concentration, and that a relatively high concentration (1.0 mg/mL) of hyaluronic acid significantly enhances the chondrogenic differentiation phenomenon at 21 days of culture. Hyaluronic acid has two major advantages on in vitro chondrogenesis; physiochemical and pharmacological effects. The physical effect of hyaluronic acid may be important, and there is evidence that the concentration of hyaluronic acid affects its rheological property [13]. Therefore, we speculated that the change in hyaluronic acid concentration altered the rheological response and the physiochemical microenvironment of cultured cells, which subsequently influenced cell differentiation.

In addition, previous studies reported that hyaluronic acid affects gene expression of type-2 collagen and aggrecan, proteoglycan synthesis, in vitro and in vivo, depending on its concentration [14-20]. Allemann et al. [15] studied the effects of hyaluronic acid added to three-dimensional scaffold on bovine chondrocyte function in vitro and reported that a high concentration of hyaluronic acid inhibited the cellularity and matrix accumulation. Akmal et al. [14] reported that supplementation of a low concentration of hyaluronic acid enhanced the glycosaminoglycan synthesis in cultured chondrocytes. Frean et al. [17] demonstrated that the effect of hyaluronic acid on proteoglycan synthesis showed a bell-shaped dose response and a higher concentration of hyaluronic acid lost a stimulatory effect on matrix production. On the other hand, Schwartz et al. [20] reported that hyaluronic acid has the dose-dependent beneficial effect on chondrogenesis by bone marrow-derived mesenchymal stem cells cultured on chitosan scaffolds. Karna et al. [18] reported that a higher concentration of hyaluronic acid increased collagen biosynthesis of human chondrocytes. These studies concluded, however, that hyaluronic acid has an optimal concentration for positive effect on chondrogenesis, although the molecular weight or concentration of hyaluronic acid varied widely among studies. However, this study is of value because it demonstrated that as the cells proliferate and differentiate, the hyaluronic acid synergistically enhances the chondrogenic effect of the PAMPS/PDMAAm DN gel in the culture condition used in this study.

The biological principle of the innovative strategy with the PAMPS/PDMAAm DN gel implantation for cartilage repair is based on recruiting cells derived from either the bone marrow or the synovium or both. Therefore, this DN gel must be a safe biomaterial for clinical application. Previous in vivo studies showed that the mechanical properties of the PAMPS/PDMAAm DN gel implanted in the subcutaneous tissue did not deteriorate at 6 weeks after implantation [10] and that although a pellet implantation test into the para-vertebral muscle induced a mild cell infiltration at 1 week, the degree of the inflammation significantly decreased to the same degree as that of the negative control at 4 and 6 weeks [21]. Furthermore, the shape or the position of the PAMPS/PDMAAm DN gel implanted in the bone defect was not much changed at 12 weeks after surgery compared to that at 4 weeks [2]. We also cultured ATDC5 cells on the PAMPS/PDMAAm DN gel [1] as well as the single-network PAMPS and PDMAAm gels [4,5]. No harmful effects due to these gel surfaces were detected. We believe that the PAMPS/PDMAAm DN gel is a biocompatible material.

There are some limitations in this study. First, we used ATDC5 cells, which are known to undergo chondrogenic differentiation under stimulation with insulin as many previous studies have used this cell line as an in vitro model for chondrogenesis. However, these cells derived from a teratocarcinoma cell-line may behave differently from native chondorocytes. In addition, we cultured the cells for only 21 days. As the period for cell differentiation depends on the type of cell, a longer term culture may show different results. Secondly, we did not evaluate chondrogenic protein expression such as glycosaminoglycan production by the cells cultured on the DN gel. Alcian blue staining may be a good method for this analysis, however, the gel itself is markedly stained by alcian blue and it is quite difficult to wash this stain completely out of the gel. Therefore, we evaluated type-2 collagen expression using a fluorescent immunocytochemical method in addition to the PCR analyses for type-2 collagen, aggrecan, and Sox9 in the cultured ATDC5 cells. Thirdly, we did not clarify the effect of hyaluronic acid on other culture systems including different cells. Thus, further studies will be needed to clarify the effect of hyaluronic acid supplementation on chondrogenic differentiation induced not only by the PAMPS/PDMAAm DN gel but also other synthetic materials. However, the present study has provided important evidence that in vitro hyaluronic acid supplementation can significantly modify the chondrogenic differentiation induced by the PAMPS/PDMAAm DN gel, depending on the concentration.

## Conclusions

The PAMPS/PDMAAm DN gel induced chondrogenic differentiation of ATDC5 cells without insulin. This effect was significantly affected by hyaluronic acid, depending on the level of concentration; a relatively high concentration (1.0 mg/mL) of hyaluronic acid significantly increased the expression of cartilage markers at 21 days of culture.

## Abbreviations

AMPS: 2-acrylamido-2-methyl-1-propanesulfonic acid; ANOVA: Analysis of variance; DMAAm: N,N’-dimethyl acrylamide; DN: Double-network; GAPDH: Glyceraldehyde-3-phosphate dehydrogenase; HA: Hyaluronic acid; MBAA: N,N^’^-methylenebisacrylamide; PAMPS: Poly-(2-Acrylamido-2-methylpropanesulfonic acid); PBS: Phosphate-buffered saline; PCR: Polymerase chain reaction; PDMAAm: Poly-(N,N’-dimethyl acrylamide); PS: Polystyrene (dish); UV: Ultraviolet.

## Competing interests

We have no financial or non- financial competing interests. We do not hold or are not currently applying for any patents relating to the content of the manuscript.

## Authors’ contribution

NK conducted all experiments and was responsible for data collection, analysis and drafting the manuscript. TF and KY were involved in study design, data analysis and edited the manuscript. TK and JPG created the DN-gel material. All authors read and approved the final manuscript.

## Pre-publication history

The pre-publication history for this paper can be accessed here:

http://www.biomedcentral.com/1471-2474/15/222/prepub
